# Prospective evaluation of patient-reported outcomes of invisible ink tattoos for the delivery of external beam radiation therapy: the PREFER trial

**DOI:** 10.3389/fonc.2024.1374258

**Published:** 2024-03-25

**Authors:** Camille Hardy-Abeloos, Daniel Gorovets, Aurora Lewis, Wenyan Ji, Alicia Lozano, Chih Chun Tung, Francis Yu, Alexandra Hanlon, Haibo Lin, Anh Kha, Yoshiya Yamada, Rafi Kabarriti, Stanislav Lazarev, Shaakir Hasan, Arpit M. Chhabra, Charles B. Simone, J. Isabelle Choi

**Affiliations:** ^1^ Department of Radiation Oncology, NYU School of Medicine, New York, NY, United States; ^2^ Department of Radiation Oncology, Memorial Sloan Kettering Cancer Center, New York, NY, United States; ^3^ New York Proton Center, New York, NY, United States; ^4^ Rutgers Robert Wood Johnson Medical School, Newark, NJ, United States; ^5^ Center for Biostatistics and Health Data Science, Department of Statistics, Virginia Tech, Roanoke, VA, United States; ^6^ Department of Radiation Oncology, Montefiore Medical Center, Bronx, NY, United States; ^7^ Department of Radiation Oncology, Mount Sinai Health System, New York, NY, United States

**Keywords:** proton therapy, radiation therapy, radiation tattoos, quality of life, image guided radiotherapy

## Abstract

**Introduction:**

Invisible ink tattoos (IITs) avoid cosmetic permanence of visible ink tattoos (VITs) while serving as more reliable landmarks for radiation setup than tattooless setups. This trial evaluated patient-reported preference and feasibility of IIT implementation.

**Methods and materials:**

In an IRB-approved, single institution, prospective trial, patients receiving proton therapy underwent IIT-based treatment setup. A survey tool assessed patient preference on tattoos using a Likert scale. Matched patients treated using our institutional standard tattooless setup were identified; treatment times and image guidance requirements were evaluated between tattooless and IIT-based alignment approaches. Distribution differences were estimated using Wilcoxon rank-sum tests or Chi-square tests.

**Results:**

Of 94 eligible patients enrolled, median age was 58 years, and 58.5% were female. Most common treatment sites were breast (18.1%), lung (17.0%) and pelvic (14.9%). Patients preferred to receive IITs versus VITs (79.8% pre-treatment and 75.5% post-treatment, respectively). Patients were willing to travel farther from home to avoid VITs versus IITs (p<0.01). Females were willing to travel (45.5% vs. 23.1%; p=0.04) and pay additional money to avoid VITs (34.5% vs. 5.1%; p<0.01). Per-fraction average +treatment time and time from on table/in room to first beam were shorter with IIT-based vs. tattooless setup (12.3min vs. 14.1min; p=0.04 and 24.1min vs. 26.2min; p=0.02, respectively).

**Discussion:**

In the largest prospective trial on IIT-based radiotherapy setup to date, we found that patients prefer IITs to VITs. Additionally, IIT-based alignment is an effective and efficient strategy in comparison with tattooless setup. Standard incorporation of IITs for patient setup should be strongly considered.

## 1 Introduction

Reproducibility in treatment setup is imperative to ensure precision and accuracy of external beam radiation therapy (EBRT) delivery. EBRT patient setup is traditionally accomplished using multiple 3- to 5-point permanent, visible ink tattoos (VITs) administered using a small needle and black or dark blue permanent ink serving as setup or isocenter markers.

Although VITs are used in standard practice for daily radiotherapy (RT) alignment, they remain long after treatment completion-indefinitely-serving as an indelible and often distressing reminder of their disease and treatment. In a recent study, 78% of women stated they would choose treatment that avoided tattoos and/or marks, even if additional efforts were required, including added cost, distance, or travel time (1). Moreover, VITs have been shown to cause significant long-term emotional distress (2–4). Reliable, high-quality alternatives to VITs are also needed for pragmatic concerns of difficulty differentiating VITs from hyperpigmented dermal lesions and challenges identifying VITs in patients with dark skin tones. An alternative that can be utilized across skin tones and freckling is necessary to ensure equitable access to optimal RT setup and delivery.

Recognizing the negative impact of visible permanent ink tattoos on patient quality of life (QoL), alternative approaches are under study, including tattooless setup and the use of alternative inks such as temporary tattoos (NCT05248009) or IITs (5–7). Validating the feasibility of these methods is imperative to ensure treatment setup accuracy and efficiency, while also systematically assessing patient preference in pursuing VITs alternatives and their impact on QoL. In an effort to avoid VITs, patients at our institution were initially aligned using a semi-permanent marker-based, tattooless alignment procedure. Given the sensitivity of intensity-modulated proton therapy (IMPT) dosimetric accuracy and robustness to changes in treatment path density, a high level of precision and reproducibility of patient setup is necessary. IITs were introduced in an attempt to improve setup accuracy and efficiency while maintaining a patient-centered focus on cosmesis preservation and long-term emotional well-being. This trial prospectively evaluated patient-reported preference on tattoo use and feasibility of implementation of IITs in a radiation oncology clinic.

## 2 Methods and materials

This prospective feasibility trial received institutional review board approval (IRB #XX). Eligibility included patients ≥18 years old undergoing proton RT at XX to any site, excluding treatments involving RT to the brain or head and neck, where tattoos are typically not applied and alignment is indexed on immobilization devices. Informed consent was obtained from all enrolled patients.

Power for this study was determined *a priori* and based on the primary endpoint of feasibility and safety of implementing IITs for RT setup and delivery. Using Simon’s two-stage design (8, 9), if <5 patients enrolled in the first stage (n=53) had a soft adverse event, then accrual would proceed to the second stage (n=45). With 98 total patients, this design achieved 80% power to detect a 4% difference in the acceptability rates (4% acceptable, 8% unacceptable) assuming a one-sided type 1 error rate of 0.20. A total of 102 patients were planned for enrollment to obtain an evaluable group of 98 patients assuming a 4% attrition rate. Ultimately, 94 patients were enrolled, after which time the trial was closed since feasibility was demonstrated and IITs became standard of cancer and ubiquitously employed at our institution. All 94 patients completed all study questionnaires, resulting in 75% power for the primary endpoint of feasibility.

### 2.1 Study interventions

**Figure 1:**
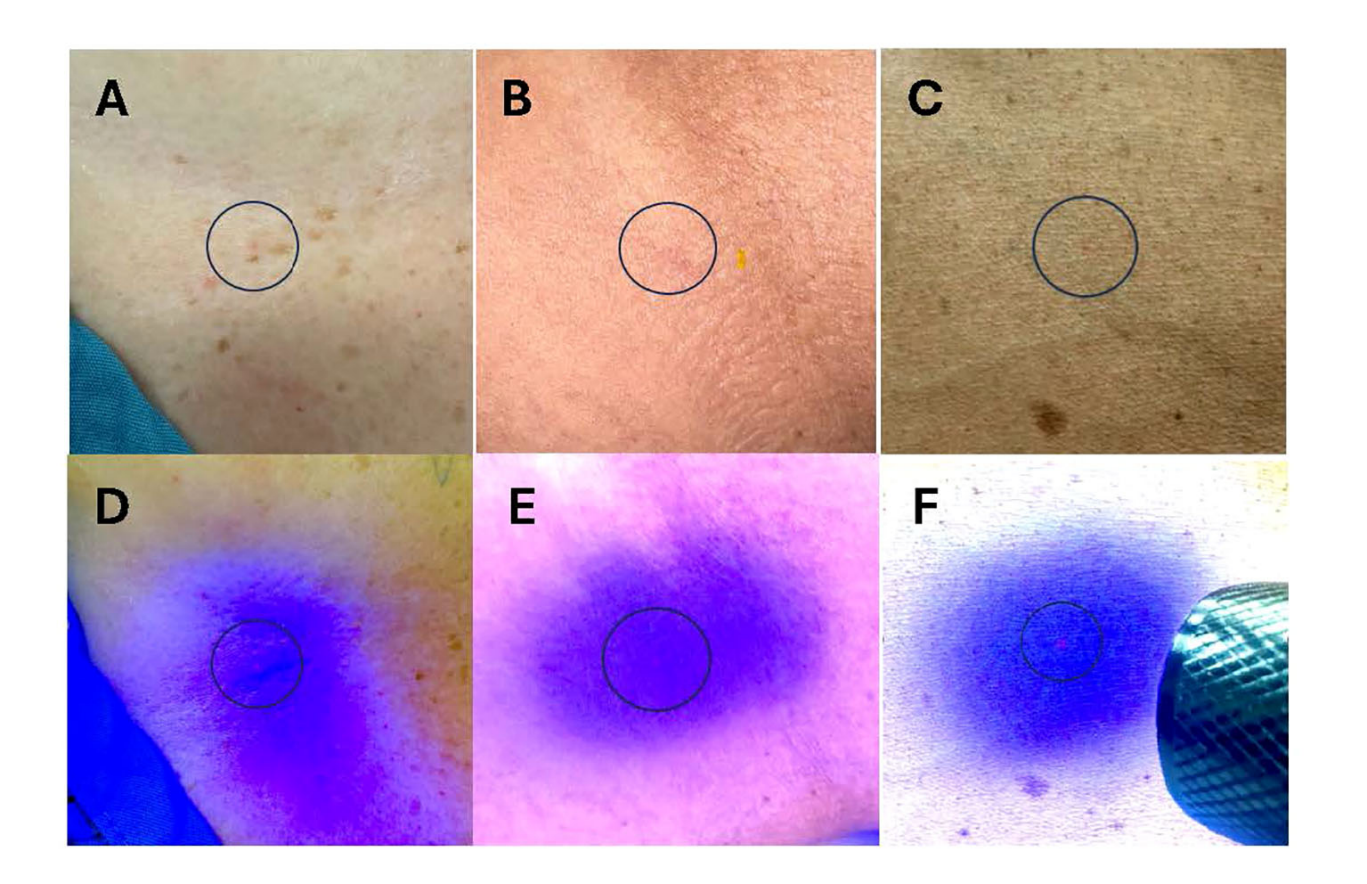
Representative photos of administered Invisible Ink Tattoos (IITs). **(A–C)** IIT under fluorescent light administered in patients with lower **(A, B)** and higher **(C)** baseline skin pigmentation. **(D–F)** IIT under blacklight flashlight in patients with lower **(D, E)** and higher **(F)** baseline skin pigmentation. Blue circles denote IIT location.

During CT simulation using IITs, patients were positioned using immobilization devices per institutional standard. Positioning was verified using AP/lateral topograms and mini-CT scans. Skin was prepped and cleaned using an alcohol swab. Qfix® Ink Align™ (Qfix, Avondale, PA) hypo-allergenic, sterile, non-toxic permanent invisible ink tattoos were then placed via a sterile needle at pre-specified treatment site locations (breast=5; abdomen=5 [8 with compression belt]; craniospinal irradiation [CSI]=9; lung/thoracic=5; pelvis=4). The Qfix® Black light flashlight was then used to verify tattoo locations. Tattoo placement was recorded and representative photos documented (**Figure 1**).

For first-fraction IIT treatment setup, the CT Simulation summary note was referenced and IITs localized using the Qfix® Black light. Patients were then aligned to IITs using treatment room lasers. Crosshairs were drawn with green marker over IITs, denoting CT marking, over which waterproof stickers were placed. Shifts were made to isocenter, and confirmatory kilovoltage (KV) x-ray images were obtained, after which treatment was delivered. Black marker then demarcated the isocenter. For subsequent treatments, patients were manually aligned to IITs, tables shifted to the isocenter, and alignments verified with KV images. IITs were also used as immutable reference points when assessing potential changes in patient anatomy or challenges in treatment alignment to inform the need for a verification scan or other intervention during the treatment course.

For our long-standing institutional standard of tattooless setups, at CT simulation, patients were positioned and immobilized, with positioning verified using AP/lateral topograms and mini-CT scans. Skin was prepped and cleaned using an alcohol swab. Green semi-permanent marker crosshairs denoted CT landmarks (breast=5; abdomen=3 [6 with compression belt]; CSI=9; lung/thoracic=5; pelvis=4). Waterproof stickers were placed on top of the marks, and mark placement recorded with representative photos documented. Wide angle photos were specifically utilized for tattooless setup to account for the likely event that one or more marks would not be visible by the first treatment.

For first-fraction tattooless treatment setup, the CT Simulation summary note was referenced to green CT marks localized if still visible. Patients were then aligned to the identified marks using treatment room lasers. Shifts were made to isocenter, and KV x-ray images confirmed patient alignment. If green marks were not visible, patients would be aligned to the vicinity of the CT marks referencing CT simulation photos, shifts applied, and images obtained to identify the isocenter. Treatment was delivered, and black semi-permanent ink demarcated the isocenter. For subsequent treatments, patients were aligned to isocenter, and alignments verified with KV images.

Enrolled patients were matched with control patients who received tattooless proton RT alignment in a 1:1 fashion according to disease site, number of treatment fields, and total number of delivered fractions. Treatment parameters recorded and compared between patients receiving IIT-based and tattooless treatment included: 1) number of KVs obtained prior to first beam during the first fraction, 2) average number of KVs obtained prior to first beam per fraction over the treatment course, 3) number of KVs obtained overall for the first fraction, 4) average number of KVs obtained per fraction over the treatment course, 5) time from on table/in room to first beam for the first fraction, 6) average time from on table/in room (defined as the time that the treatment plan was loaded) to first beam per fraction over the treatment course, 7) total treatment time for the first fraction (defined as first beam on to completion of final beam delivery), and 8) average total treatment time per fraction over the treatment course.

Patient surveys assessing preference comparing VITs and IITs, experience of receiving IITs, and cosmesis of IITs were completed prior to simulation (17 questions), immediately following simulation (5 questions), and at treatment completion (18 questions), with preference and experience scored on a 5-point Likert scale from strongly disagree to strongly agree and cosmesis scored on a 4-point Likert scale of excellent-good-fair-poor.

### 2.2 Statistical analysis

Descriptive statistics were used to characterize patient demographics (age, gender, race, ethnicity, distance from home to treatment center) and clinical characteristics (treatment site, diagnosis). Categorical variables were described with frequencies and percentages. Continuous variables were described with means/medians and interquartile ranges. Differences in distributions between trial and matched patients were estimated using Wilcoxon rank-sum tests or Chi-square tests, as appropriate.

Pre- and post- treatment questionnaires were summarized using frequencies and percentages overall and by gender. Pre- and post- treatment differential distributions were compared using symmetry tests given repeated measures within the same patients. For comparison of differences in distributions between males and females pre- and post-treatment, Chi-square tests or Fisher’s exact tests were used, as appropriate. Generalized estimating equations models were used to examine associations between traveling 0 miles versus >0 miles or paying $0 versus >$0 to avoid receiving IITs versus VITs within each treatment group (pre-treatment/post-treatment).

End-of-treatment questionnaires were summarized using frequencies and percentages. Treatment setup characteristics were summarized using means, medians, interquartile ranges, and ranges. Differences in medians between IITs versus tattooless groups relied on Wilcoxon rank-sum tests.

All statistical analyses were performed using SAS 9.4 (SAS Institute Inc., Cary, NC), with R Statistical Software (version 4.3.1) to generate data visualizations. Statistical significance was defined as p<0.05 to assess secondary endpoints not accounting for multiplicity.

## 3 Results

Between 09/2021-04/2022, 106 patients were screened, of whom 5 declined consent and 7 failed screening due to non-English primary language. In total, 94 patients enrolled on the trial, all of whom completed pre-simulation, post-simulation, and end-of-treatment patient surveys (100% response rate at all timepoints).

The median age was 58 years old (IQR: 40-68 years old) (**Table 1**). The majority were female (58.5%), White (78.7%), not of Hispanic origin (79.8%) and lived <50 miles from the treatment center (64.9%). Among both cases and controls, the most common disease sites were breast (18.1%), lung (17.0%) and pelvis (14.9%). Cases were also well-matched to controls according to number of fractions and number of treatment fields.

**Table 1:** Patient demographics and clinical characteristics.

Characteristics	Enrolled patients (N= 94)	Matched patients (N=94)	*p-*value*
**Median age (years) (IQR)**	58 (40-68)	59.5 (49-72)	0.15
Gender, n (%)			0.66
Female	55 (58.5%)	58 (61.7%)	
Male	39 (41.5%)	36 (38.3%)	
Race, n (%)			<0.01
African American	13 (13.8%)	8 (8.5%)	
White	74 (78.7%)	59 (62.8%)	
Asian	5 (5.3%)	9 (9.6%)	
Other	0 (0%)	8 (8.5%)	
Decline to Answer	2 (2.1%)	10 (10.6%)	
Ethnicity, n (%)			<0.01
Hispanic/Latino/Spanish Origin	6 (6.4%)	22 (23.4%)	
Not Hispanic/Latina/Spanish Origin	75 (79.8%)	60 (63.8%)	
Decline to Answer	13 (13.8%)	12 (12.8%)	
Disease Site, n (%)			>0.99
Breast	17 (18.1%)	17 (18.1%)	
Lung	16 (17.0%)	16 (17.0%)	
Pelvis	14 (14.9%)	14 (14.9%)	
Chest wall	12 (12.8%)	12 (12.8%)	
Prostate	11 (11.7%)	11 (11.7%)	
CSI	8 (8.5%)	8 (8.5%)	
Liver	5 (5.3%)	5 (5.3%)	
Rectum	3 (3.2%)	3 (3.2%)	
Other	8 (8.5%)	8 (8.5%)	
**Average Number of Fields (IQR)**	3.21 (3-4)	3.15 (2-4)	0.66
**Average Number of Fractions (IQR)**	17.94 (5-26)	18.47 (5-27)	0.75
Distance from Home to NYPC, n (%)			
< 20 miles	29 (30.9%)		
20-50 miles	32 (34.0%)		
50-100 miles	18 (19.1%)		
> 100 miles	11 (11.7%)		
Other	4 (4.3%)		

*P-values were calculated based on Wilcoxon Rank-sum tests or Chi-square test, as appropriate.

No patient experienced an adverse or unexpected reaction from IITs (no swelling, persistent erythema, infection). Similar patient survey responses were observed pre- and post-treatment (**Table 2**). Most patients preferred to receive IITs over VITs (79.8% at pre-treatment, 75.5% at post-treatment, p=0.11). Of 56 pre-treatment and 54 post-treatment patients stating the primary reason for their preference to avoid VITs, 69.6% and 72.2% stated that visibility/aesthetics was their primary concern, respectively. Emotional distress (17.9% pre-treatment, 18.5% post-treatment) and pain (5.4% pre-treatment, 9.3% post-treatment) were also cited as reasons to avoid VITs.

**Table 2:** Patient preference questionnaire responses pre- and post-treatment (N=94).

Question	Response	Pre-treatment	Post-treatment	*p-*value*
I would prefer not to receive any tattoo for my radiation treatment.	Agree	20 (21.3%)	21 (22.3%)	0.24
	Neutral	24 (25.5%)	33 (35.1%)	
	Disagree	50 (53.2%)	40 (42.6%)	
If I have to receive tattoos for my radiation treatment, I prefer to receive invisible tattoos.	Agree	75 (79.8%)	71 (75.5%)	0.11
	Neutral	19 (20.2%)	17 (18.1%)	
	Disagree	0 (0.0%)	6 (6.4%)	
If you would prefer not to receive visible tattoos, what is the primary reason?	Emotional Distress	10 (17.9%)	10 (18.5%)	0.49
	Pain of Tattoo Placement	3 (5.4%)	5 (9.3%)	
	Visibility/Aesthetics	39 (69.6%)	39 (72.2%)	
	Other	4 (7.1%)	0 (0.0%)	
Please note additional reasons you would prefer not to receive visible tattoos.	Emotional Distress	15 (51.7%)	13 (37.1%)	0.66
	Pain of Tattoo Placement	3 (10.3%)	7 (20.0%)	
	Visibility/Aesthetics	10 (34.5%)	13 (37.1%)	
	Other	1 (3.4%)	2 (5.7%)	
If you would prefer not to receive invisible tattoos, what is the primary reason?	Emotional Distress	1 (4.8%)	2 (11.1%)	0.97
	Pain of Tattoo Placement	12 (57.1%)	10 (55.6%)	
	Visibility/Aesthetics	3 (14.3%)	3 (16.7%)	
	Other	5 (23.8%)	3 (16.7%)	
Please note additional reasons you would prefer not to receive invisible tattoos.	Emotional Distress	1 (25.0%)	0 (0.0%)	–
	Pain of Tattoo Placement	1 (25.0%)	0 (0.0%)	
	Visibility/Aesthetics	0 (0.0%)	1 (25.0%)	
	Other	2 (50.0%)	3 (75.0%)	
Regarding VISIBLE tattoos: I would be willing to travel from my home in order to avoid receiving visible tattoos:	0 miles	53 (56.4%)	60 (63.8%)	0.64
	> 20 miles	21 (22.3%)	12 (12.8%)	
	> 35 miles	7 (7.4%)	8 (8.5%)	
	> 50 miles	8 (8.5%)	12 (12.8%)	
	> 100 miles	5 (5.3%)	2 (2.1%)	
Regarding VISIBLE tattoos: I would be willing to pay additional money in order to avoid receiving visible tattoos:	$0	78 (83.0%)	73 (77.7%)	0.83
	> $500	11 (11.7%)	16 (17.0%)	
	> $1,000	2 (2.1%)	5 (5.3%)	
	> $5,000	2 (2.1%)	0 (0.0%)	
	> $15,000	1 (1.1%)	0 (0.0%)	
Regarding INVISIBLE tattoos: I would be willing to travel from my home in order to avoid receiving invisible tattoos:	0 miles	83 (88.3%)	84 (89.4%)	0.90
	> 20 miles	5 (5.3%)	5 (5.3%)	
	> 35 miles	4 (4.3%)	1 (1.1%)	
	> 50 miles	2 (2.1%)	4 (4.3%)	
Regarding INVISIBLE tattoos: I would be willing to pay additional money in order to avoid receiving invisible tattoos:	$0	88 (93.6%)	92 (98.9%)	0.07
	> $500	6 (6.4%)	0 (0.0%)	
	> $1000	0 (0.0%)	1 (1.1%)	

*P-values were calculated based on symmetry tests. The p-value of variable “Please note additional reasons you would prefer not to receive invisible tattoos.” cannot be computed due to insufficient observations.

Pre-treatment and post-treatment, patients were 5.8 (OR=5.84, 95% CI=3.06-11.13, p<0.01) and 4.8 (OR=4.76, 95% CI=2.47-9.18, p<0.01) times more likely to travel farther away from their homes to avoid VITs versus IITs, respectively (**Table 3**). Patients were 3.0 times (OR=3.01, 95% CI=1.25-7.22, p=0.01) and 25.5 times (OR=25.46, 95% CI=3.82-169.90, p<0.01) more likely to pay additional money to avoid receiving VITs versus IITs pre-treatment and post-treatment, respectively.

**Table 3:** Generalized Estimating Equations (GEE) models of travel distance and money payment per tattoo group (N=94).

Treatment	Outcome	Tattoo Group	Odds Ratio (95% CI)	*p*-value
Pre-treatment	Travel Distance (0 vs. >0 miles)	avoid receiving invisible tattoos vs. avoid receiving visible tattoos (REF)	5.84 (3.06, 11.13)	<0.01
	Money Payment ($0 vs. >$0)	avoid receiving invisible tattoos vs. avoid receiving visible tattoos (REF)	3.01 (1.25, 7.22)	0.01
Post-treatment	Travel Distance (0 vs. >0 miles)	avoid receiving invisible tattoos vs. avoid receiving visible tattoos (REF)	4.76 (2.47, 9.18)	<0.01
	Money Payment ($0 vs. >$0)	avoid receiving invisible tattoos vs. avoid receiving visible tattoos (REF)	25.46 (3.82, 169.90)	<0.01

REF, Reference; GEE, Generalized Estimating Equations.

Assessing survey responses pre- and post- treatment by gender, more females than males preferred to receive IITs vs. VITs (pre-treatment: 87.3% vs. 69.2%, p=0.03; post-treatment: 89.1% vs. 56.4%, p<0.01) (**Table 4**). For both males and females, the primary reason to avoid VITs was visibility/aesthetics. More females than males were willing to travel farther from their homes to avoid receiving VITs (pre-treatment: 58.2% vs. 23.1%, p=0.01; post-treatment: 45.5% vs. 23.1%, p=0.04). More females were willing to pay additional money to avoid VITs in the post-treatment questionnaire (34.5% vs. 5.1%, p<0.01).

**Table 4:** Patient preference questionnaire responses pre- and post-treatment by gender (N=94).

		Pre-treatment, n (%)			Post-treatment, n (%)		
		Female (N =55)	Male (N=39)	*p-*value*	Female (N=55)	Male N=39)	*p-*value*
I would prefer not to receive any tattoo for my radiation treatment.	Agree	15 (27.3%)	5 (12.8%)	0.21	16 (29.1%)	5 (12.8%)	0.05
	Neutral	14 (25.5%)	10 (25.6%)		21 (38.2%)	12 (30.8%)	
	Disagree	26 (47.3%)	24 (61.5%)		18 (32.7%)	22 (56.4%)	
If I have to receive tattoos for my radiation treatment, I prefer to receive invisible tattoos.	Agree	48 (87.3%)	27 (69.2%)	0.03	49 (89.1%)	22 (56.4%)	<0.01
	Neutral	7 (12.7%)	12 (30.8%)		4 (7.3%)	13 (33.3%)	
	Disagree	0 (0.0%)	0 (0.0%)		2 (3.6%)	4 (10.3%)	
If you would prefer not to receive visible tattoos, what is the primary reason?	Emotional Distress	9 (23.7%)	1 (5.6%)	0.17	9 (23.7%)	1 (6.3%)	0.28
	Pain of Tattoo Placement	3 (7.9%)	0 (0.0%)		4 (10.5%)	1 (6.3%)	
	Visibility/Aesthetics	24 (63.2%)	15 (83.3%)		25 (65.8%)	14 (87.5%)	
	Other	2 (5.3%)	2 (11.1%)		0 (0.0%)	0 (0.0%)	
Please note additional reasons you would prefer not to receive visible tattoos.	Emotional Distress	15 (62.5%)	0 (0.0%)	<0.01	12 (41.4%)	1 (16.7%)	0.38
	Pain of Tattoo Placement	1 (4.2%)	2 (40.0%)		6 (20.7%)	1 (16.7%)	
	Visibility/Aesthetics	8 (33.3%)	2 (40.0%)		10 (34.5%)	3 (50.0%)	
	Other	0 (0.0%)	1 (20.0%)		1 (3.4%)	1 (16.7%)	
If you would prefer not to receive invisible tattoos, what is the primary reason?	Emotional Distress	1 (5.9%)	0 (0.0%)	0.35	2 (16.7%)	0 (0.0%)	0.19
	Pain of Tattoo Placement	11 (64.7%)	1 (25.0%)		8 (66.7%)	2 (33.3%)	
	Visibility/Aesthetics	2 (11.8%)	1 (25.0%)		1 (8.3%)	2 (33.3%)	
	Other	3 (17.6%)	2 (50.0%)		1 (8.3%)	2 (33.3%)	
Please note additional reasons you would prefer not to receive invisible tattoos.	Emotional Distress	1 (25.0%)	0 (0.0%)	–	0 (0.0%)	0 (0.0%)	>0.99
	Visibility/Aesthetics	0 (0.0%)	0 (0.0%)		1 (33.3%)	0 (0.0%)	
	Pain of Tattoo Placement	1 (25.0%)	0 (0.0%)		0 (0.0%)	0 (0.0%)	
	Other	2 (50.0%)	0 (0.0%)		2 (66.7%)	1 (100.0%)	
Regarding VISIBLE tattoos: I would be willing to travel from my home in order to avoid receiving visible tattoos:	0 miles	23 (41.8%)	30 (76.9%)	<0.01	30 (54.5%)	30 (76.9%)	0.04
	> 20 miles	15 (27.3%)	6 (15.4%)		7 (12.7%)	5 (12.8%)	
	> 35 miles	7 (12.7%)	0 (0.0%)		8 (14.5%)	0 (0.0%)	
	> 50 miles	6 (10.9%)	2 (5.1%)		9 (16.4%)	3 (7.7%)	
	> 100 miles	4 (7.3%)	1 (2.6%)		1 (1.8%)	1 (2.6%)	
Regarding VISIBLE tattoos: I would be willing to pay additional money in order to avoid receiving visible tattoos:	$0	41 (74.5%)	37 (94.9%)	0.09	36 (65.5%)	37 (94.9%)	<0.01
	> $500	9 (16.4%)	2 (5.1%)		14 (25.5%)	2 (5.1%)	
	> $1,000	2 (3.6%)	0 (0.0%)		5 (9.1%)	0 (0.0%)	
	> $5,000	2 (3.6%)	0 (0.0%)		0	0 (0.0%)	
	> $15,000	1 (1.8%)	0 (0.0%)		0 (0.0%)	0 (0.0%)	
Regarding INVISIBLE tattoos: I would be willing to travel from my home in order to avoid receiving invisible tattoos:	0 miles	48 (87.3%)	35 (89.7%)	0.94	48 (87.3%)	36 (92.3%)	0.23
	> 20 miles	3 (5.5%)	2 (5.1%)		3 (5.5%)	2 (5.1%)	
	> 35 miles	3 (5.5%)	1 (2.6%)		0 (0.0%)	1 (2.6%)	
	> 50 miles	1 (1.8%)	1 (2.6%)		4 (7.3%)	0 (0.0%)	
Regarding INVISIBLE tattoos: I would be willing to pay additional money in order to avoid receiving invisible tattoos:	$0	51 (92.7%)	37 (94.9%)	> 0.99	53 (98.1%)	39 (100.0%)	>0.99
	> $500	4 (7.3%)	2 (5.1%)		0 (0.0%)	0 (0.0%)	
	> $1,000	0 (0.0%)	0 (0.0%)		1 (1.9%)	0 (0.0%)	

*P-values were calculated based on Chi-square tests or Fisher’s exact tests, as appropriate. For variable “Please note additional reasons you would prefer not to receive invisible tattoos,” *p*-value could not be determined due to lack of patients in male group.

**Figure 2:**
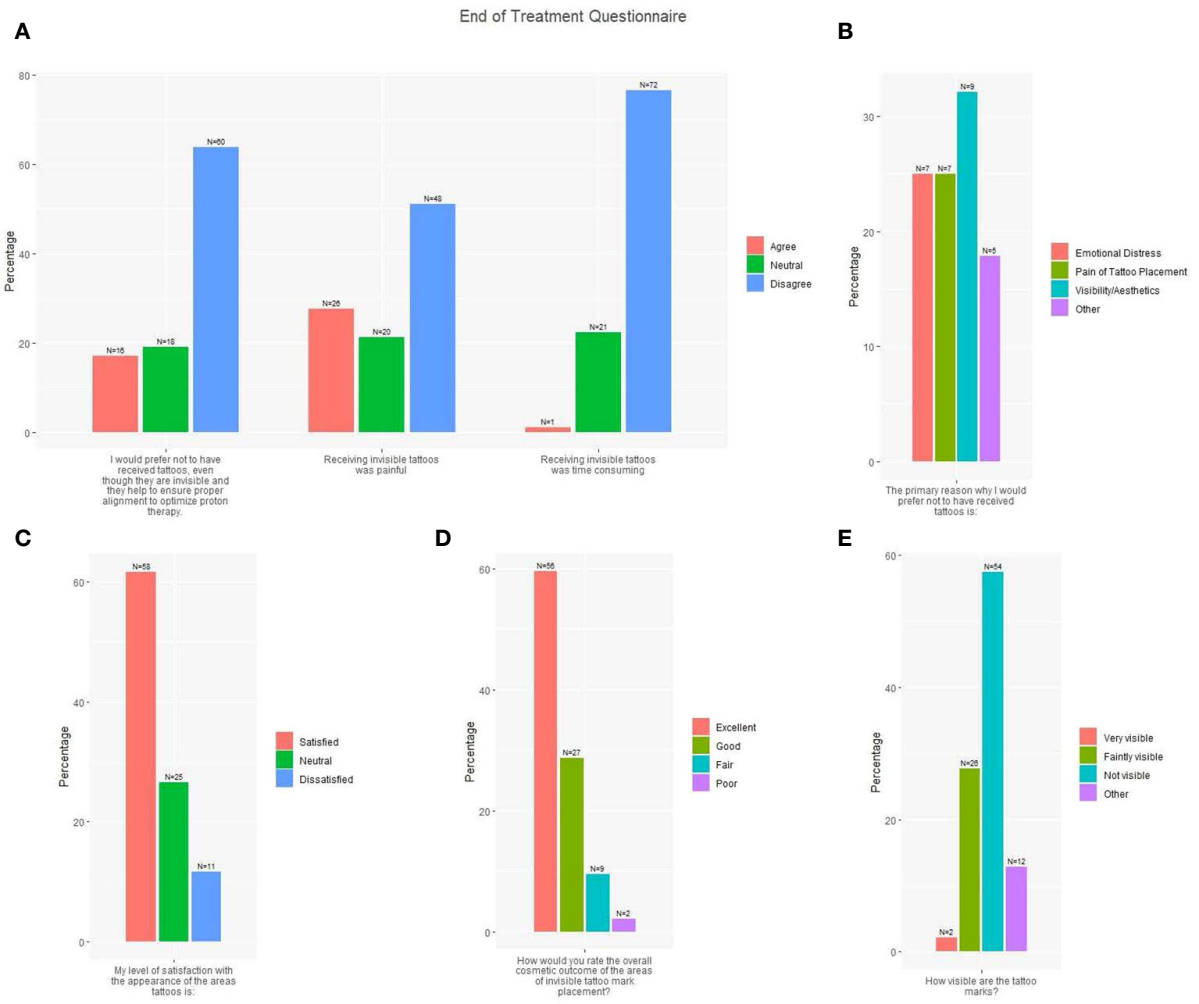
End of treatment questionnaire responses (N=94). **(A)** Three survey questions with responses: I would prefer not to have received tattoos, even though they are invisible and they help to ensure proper alignment to optimize proton therapy; Receiving invisible tattoos was painful; Receiving invisible tattoos was time consuming (Red = Agree; Green = Neutral; Blue = Disagree). **(B)** One survey question with response: The primary reason why I would prefer not to have received tattoos is X (Red = Emotional Distress; Green = Pain of Tattoo Placement; Blue = Visibility/Aesthetics; Purple = Other). **(C)** One survey question with response: My level of satisfaction with the appearance of the areas tattooed is X (Red = Satisfied; Green = Neutral; Blue = Dissatisfied). **(D)** One survey question with response: How would you rate the overall cosmetic outcome of the areas of invisible tattoo mark placement? (Red = Excellent; Green = Good; Blue = Fair; Purple = Poor). **(E)** One survey question with response: How visible are the tattoo marks? (Red = Very visible; Green = Faintly visible; Blue = Not visible; Purple = Other).

At treatment completion, 88.3% of patients rated overall cosmesis of IITs as excellent or good (**Figure 2**). Only 2.1% of patients rated their tattoo marks as visible; 27.7% rated them as faintly visible, 57.4% as not visible, and 12.8% as other including “unsure” or “can’t tell.” Most patients (61.7%) were satisfied with the appearance of their tattoos, 27.7% reported IIT receipt was painful, and one patient (1.1%) reported IIT placement was time-consuming. Most patients (63.8%) would prefer to receive IITs to ensure proper alignment versus no tattoos. Of 28 patients who provided a primary reason to avoid IITs, 32.1% cited visibility/aesthetics, 25.0% emotional distress, 25.0% pain of tattoo placement, and 17.9% other reasons, including “prefer not to have anything injected in my body” or “don’t want anything permanent in body”.

In a comparison of IIT trial patients and matched patients treated with tattooless alignment, average per-fraction total treatment time and average time from on table/in room to first beam was shorter with IITs compared to tattooless setup (12.29min (range: 3.00-38.00) vs. 14.10min (range: 2.85-40.10); p=0.04 and 24.13min (range: 14.00-44.00min) vs. 26.22min (range: 13.00-42.00); p=0.02, respectively) (**Table 5**). No statistically significant differences were observed for median number of KVs before first beam for first fraction and for each fraction and total number of KVs for first fraction and each fraction. Total treatment time for the first fraction (beam on time) approached significance in favor of shorter time with the use of IITs versus tattooless setup (13.10min (range: 3-109) vs. 16.80min (2-125), p=0.06).

**Table 5:** Pre-treatment imaging and treatment duration with invisible ink vs. tattooless setup.

		Invisible Ink Tattoos	Tattooless	*p*-value*
**# of KVs before first beam for first fraction**	Mean	9.79	9.16	0.16
	Median	8.00	6.00	
	IQR	6.00 - 12.00	4.00 - 10.00	
	Range	2.00 - 52.00	2.00 - 48.00	
**Average # of KVs before first beam for each fraction**	Mean	8.58	8.66	0.83
	Median	8.54	8.38	
	IQR	3.73 - 12.88	3.55 - 13.40	
	Range	1.73 - 16.89	2.00 - 17.57	
**# of KVs for first fraction (all beams)**	Mean	12.32	12.04	0.45
	Median	10.00	8.00	
	IQR	6.00 - 14.00	6.00 - 14.00	
	Range	4.00 - 60.00	2.00 - 56.00	
**Average # of KVs for each fraction (all beams)**	Mean	10.42	10.12	0.40
	Median	7.86	7.24	
	IQR	6.43 - 10.80	5.53 - 12.00	
	Range	4.48 - 42.45	2.96 - 35.60	
**Time from on table/in room to first beam on for first fraction (min)**	Mean	35.40	39.30	0.07
	Median	32.00	35.50	
	IQR	26.00 - 39.00	28.00 - 47.00	
	Range	10.00 - 106.00	19.00 - 105.00	
**Average time from on table/in room to first beam on for each fraction (min)**	Mean	24.13	26.22	0.02
	Median	23.00	26.00	
	IQR	20.00 - 27.00	21.00 - 32.00	
	Range	14.00 - 44.00	13.00 - 42.00	
**Total treatment time (beam on time) for first fraction (min)**	Mean	13.10	16.80	0.06
	Median	10.00	12.00	
	IQR	6.00 - 16.00	9.00 - 20.00	
	Range	3.00 -109.00	2.00 - 125.00	
**Average treatment time (min)**	Mean	12.29	14.10	0.04
	Median	11.34	12.95	
	IQR	7.31 - 14.04	9.16 - 18.00	
	Range	3.00 - 38.00	2.85 - 40.10	

*P-values were calculated based on Wilcoxon rank-sum tests; IQR, Interquartile Range.

## 4 Discussion

Our study demonstrated safety and feasibility of implementation of an IIT-based treatment setup procedure. Treatment efficiency in patient setup was also improved compared with our previously-utilized tattooless setup approach, with reduced per-fraction average total treatment time and time to first beam with IITs. Assessment of patient preference of tattoo-based options showed strong preference for IITs over VITs, with most patients reporting visibility/aesthetics as the primary reason for their desire to avoid VITs (40% out of entire cohort, 71% of patients who answered the survey question).

These findings are consistent with prior studies. In a non-blinded, single-center, randomized control trial by Landeg et al. of 46 breast cancer patients randomized to RT with IITs versus VITs (3), patients with VITs reported worse body image scores at 1 and 6 months post-treatment compared to baseline relative to patients receiving IITs. Comments from body image score questionnaires similarly showed concerns about visibility of VITs, including “I feel much better without tattoos being visible. Much more confident.” In a prospective, randomized control trial of 34 breast cancer patients receiving RT, Lim et al. showed that all patients with UV ink tattoos were satisfied with tattoo appearance and did not feel cautious about clothing choices versus 82.4% and 88.2%, respectively, of patients with VITs at 6 weeks post-RT (10). Furthermore, of 12 radiation therapists responding to staff satisfaction surveys, 9 commented that IITs preserve body image perception.

Our study showed patients demonstrated a willingness to travel farther from home and pay additional money to avoid VITs compared with IITs. By implementing IITs at more radiation treatment centers, equitable access to this intervention can be optimized, regardless of demographic or socioeconomic status. Our study found that women were particularly more willing to travel and pay additional money to avoid VITs. At treatment completion, more females responded preferring IITs over VITs, thus highlighting the importance of avoiding VITs in women. Lim et al. further reported on the ease of use and implementation of IITs for breast/chest wall RT (10). Most (88.2%) of radiation therapists (n=30) felt minimum effect of UV ink on the overall setup time, and 94.3% (n=33) did not notice difficulty localizing the UV ink tattoo during patient positioning. They further reported no difference in mean setup errors between IITs and VITs. Landeg et al. similarly showed no significant differences in systematic and random setup errors using electronic portal images between IITs and VITs (3). Compared to the studies by Landeg and Lim that only included breast cancer patients, our study showed that IITs can be applied to various disease sites, while retaining patient setup accuracy and treatment efficiency along with a low burden of implementation and minimal change or cost in existing clinical workflows.

Recently, surface-guided radiation therapy (SGRT) has emerged as an alternative to tattoo-based setup (11–15). Giantsoudi et al. showed no difference in setup shifts or average treatment time per fraction in patients receiving tattooless regional nodal irradiation (RNI) with volumetric modulated arc therapy (VMAT) versus those receiving three-dimensional conformal RT RNI with VITs (6). Zhao et al. showed larger rotations (>3° in any yaw, roll or pitch direction) in 18.6% of surface-guided setups, with the majority observed for abdominal and pelvic targets, highlighting the importance of checking a patient’s entire body pose (7). Naidoo et al. performed a systematic review of 13 studies evaluating the accuracy and reproducibility of patient setup with SGRT versus conventional tattoo-based methods and showed SGRT mean systematic errors in the 3 translational directions were reduced relative to tattoo setups alone, and mean rotational errors were equivalent, thereby concluding that SGRT could improve setup accuracy and efficiency (16). SGRT, however, remains a costly technology, requires additional training, workflow changes, and potential setup inefficiencies for abdominopelvic targets with large surface variations. Our trial showed that average total treatment time per fraction was shorter with IITs compared to tattooless setup, suggesting more rapid alignment for each field with IITs. Similarly, average time from on table/in room to first beam on for each fraction was significantly shorter with IIT compared to tattooless setup, again suggesting faster setup and alignment. This is consistent with and may be representative of challenges that arose with our previous tattooless treatment workflow, in which waterproof stickers and markers were used but were commonly not visible by the time of first treatment. In addition, dark ink marks may not be as readily localizable in patients with higher baseline skin pigmentation, which could also pose a barrier for routine use of a tattooless setup relying on these landmarks. IITs may, therefore, be a more cost-effective and efficient alternative to tattooless setup, while still eliminating cosmetic and emotional concerns of VITs.

Limitations of this study include enrollment terminating at 94 instead of the planned 98 evaluable patients. However, as no adverse events occurred contributing to the feasibility analysis (n=0), this number and resulting power adequately demonstrates feasibility of IIT administration. Although significant differences between comparison groups were identified, this study was not powered for secondary outcomes. In addition, our study did not include 3D vector setup shift data and had limited follow-up, with the last questionnaire administered at treatment completion. Later survey timepoints could have provided a more robust representation of patient satisfaction and emotional distress after IIT receipt. Even with this more limited follow-up, however, there was a clear patient preference to receive IITs vs. VITs.

At treatment completion, most patients were satisfied with their tattoo appearance, and 88.3% rated the overall cosmetic outcome of their IITs as excellent or good, with over half reporting that the tattoo marks were not visible. IITs implementation appears feasible and straightforward in the clinical workflow and allows for reproducible accuracy in patient positioning and treatment efficiency, while also preventing long-term negative emotional impact from a permanent visible tattoo.

In the largest prospective trial performed on IITs to date, we found that patients prefer to receive IITs versus dark VITs. Implementation of IITs was feasible and applicable to various disease sites (breast, abdomen, craniospinal axis, thorax, pelvis), while allowing for improved treatment efficiency relative to tattooless setups. In light of the potential positive impact on long-term patient QoL and emotional well-being, along with its modest cost and ease of use, standard incorporation of IITs for RT patient alignment should be strongly considered.

## Data availability statement

The original contributions presented in the study are included in the article/supplementary material. Further inquiries can be directed to the corresponding author.

## Ethics statement

The studies involving humans were approved by Western Institutional Review Board. The studies were conducted in accordance with the local legislation and institutional requirements. The participants provided their written informed consent to participate in this study.

## Author contributions

CH-A: Data curation, Formal analysis, Writing – original draft, Writing – review & editing. DG: Writing – review & editing. ALe: Data curation, Writing – review & editing. WJ: Data curation, Formal analysis, Writing – original draft, Writing – review & editing. ALo: Data curation, Formal analysis, Writing – review & editing. CT: Data curation, Writing – review & editing. FY: Data curation, Writing – review & editing. AH: Data curation, Formal analysis, Writing – review & editing. HL: Data curation, Writing – review & editing. AK: Investigation, Writing – review & editing. YY: Writing – review & editing. RK: Writing – review & editing. SL: Writing – review & editing. SH: Writing – review & editing. AC: Writing – review & editing. CS: Supervision, Writing – review & editing. JC: Conceptualization, Data curation, Formal analysis, Funding acquisition, Investigation, Supervision, Writing – original draft, Writing – review & editing.
